# Resistive switching mechanism in the one diode-one resistor memory based on p^+^-Si/n-ZnO heterostructure revealed by *in-situ* TEM

**DOI:** 10.1038/srep45143

**Published:** 2017-03-21

**Authors:** Lei Zhang, Liang Zhu, Xiaomei Li, Zhi Xu, Wenlong Wang, Xuedong Bai

**Affiliations:** 1 Beijing National Laboratory for Condensed Matter Physics and Institute of Physics, Chinese Academy of Sciences, Beijing 100190, China; 2 Collaborative Innovation Center of Quantum Matter, Beijing 100190, China; 3 School of Physical Sciences, University of Chinese Academy of Sciences, Beijing 100190, China

## Abstract

One diode-one resistor (1D1R) memory is an effective architecture to suppress the crosstalk interference, realizing the crossbar network integration of resistive random access memory (RRAM). Herein, we designed a p^+^-Si/n-ZnO heterostructure with 1D1R function. Compared with the conventional multilayer 1D1R devices, the structure and fabrication technique can be largely simplified. The real-time imaging of formation/rupture process of conductive filament (CF) process demonstrated the RS mechanism by *in-situ* transmission electron microscopy (TEM). Meanwhile, we observed that the formed CF is only confined to the outside of depletion region of Si/ZnO pn junction, and the formation of CF does not degrade the diode performance, which allows the coexistence of RS and rectifying behaviors, revealing the 1D1R switching model. Furthermore, it has been confirmed that the CF is consisting of the oxygen vacancy by *in-situ* TEM characterization.

Resistive random access memory (RRAM) has been considered to be the candidate to overcome the physical and technological limitations for next-generation nonvolatile memory due to their superior performance, such as simple structure, fast switching speed, high storage density, and low power consumption^1^,^2^,^3^,^4^. For further application of RRAMs, it is necessary to realize the integration of a mass of RRAMs. A passive crossbar array is regarded as one of the most promising architectures for RRAM integration, because of the simplicity, scalability, and multiple stackability of this structur^5^,^6^,^7^. However, the crossbar array architecture suffers from an intrinsic crosstalk problem in practical applications. Some function devices, especially one diode-one resistor (1D1R) device, can effectively suppress the crosstalk current and achieve high-level integration in the crossbar network^8^,^9^,^10^. Most of conventional 1D1R devices usually consist of a stack of sandwich-structure RRAM and pn junction diode, thus having a complex multilayer structure^11^,^12^. Compared with the traditional 1D1R devices with discrete components, some simple sandwich structures (e.g., Ti/TiO_2_/Pt, n^+^-Si/HfO_2_/Ni) have been designed to realize the 1D1R function, effectively decreasing the complexity of fabrication process and potentially increasing the 3D integratio^10^,^13^. However, the switching mechanism is still uncertain in thesesingle-stacked1D1R devices. There is less direct evidence to clarify the relationship between the resistance states and their corresponding internal structures due to the difficulty of analyzing in an oxidation state insulator. Recently, the *in-situ* transmission electron microscope (TEM) has been shown to be a powerful tool for studying the ion migration and electrochemical reactions at the nanoscale^14^,^15^,^16^. Some groups have observed the forming/rupture of conductive filament (CF) and analyzed the CF’s components and structures by electron energy loss spectroscopy (EELS) and energy dispersive spectroscopy (EDS) equipped with *in-situ* TEM^17^,^18^.

Herein, we designed a p^+^-Si/n-ZnO/Al heterostructure device. The simple stacked device shows coexistence of the rectification and resistive switching (RS) characteristics, realizing the 1D1R switching functions. Furthermore, we demonstrate that the RS is associated with the formation and rupture of CF by the *in-situ* TEM observations. The TEM images, EDS and EELS spectrum have been used to identify the CF’s components, indicating that the dominant conducting species is oxygen vacancy. Importantly, we observed the existence of pn junction during the RS processes, and the formation and rupture of localized CF model is proposed to explain the 1D1R switching behavior.

## Results and Discussion

**Figure 1:**
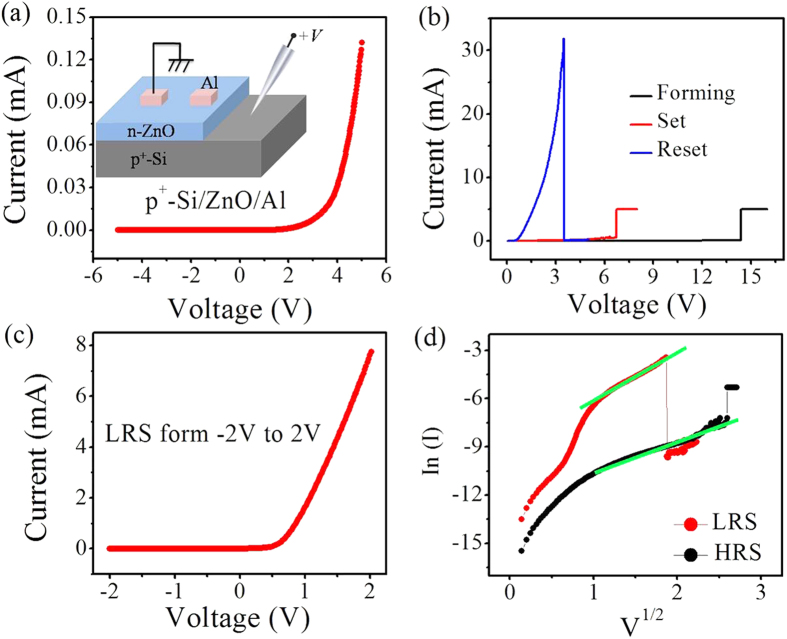
(**a**) The rectifying I-V curve for the initial p^+^-Si/n-ZnO/Al memory device. Structure diagram of this device is shown in the inset of (**a**). (**b**) The forming process and unipolar resistive switching of this 1D1R device. (**c**) LRS I-V curve with a rectifying characteristics. (**d**) Plots of V^1/2^-In(I) for the HRS/LRS of this 1D1R device. The linear fit of green line is shown for the HRS and LRS.

A structural diagram of the p^+^-Si/n-ZnO/Al 1D1R device is shown in the inset of Fig. 1(a). The heavily doped p-type Si substrate is used to form pn heterojunction, which can also be regarded as the bottom electrode for the RRAM. The ZnO film has been experimentally determined to be n-type by a negative Hall coefficient. Thus, the n-ZnO can serve as n-type semiconductor layer as well as the switching layer, realizing the 1D1R structure design. The initial heterostructure device shows the asymmetric rectifying current-voltage (I-V) characteristics, as shown in Fig. 1(a), which could attribute to the rectifying effect of the p^+^-Si/n-ZnO junction diode. To justify this, the energy band structure of p^+^-Si/n-ZnO/Al device and the I-V cures of Al/ZnO/Al reference device were shown in Figs S1 and S2 of the supporting information. A ‘forming’ process is required to activate the RS behavior by applying a positive voltage on the p^+^-Si electrode. Here, a proper current compliance (CC) of 5 mA was selected to protect the device from hard breakdown. The level of CC can change the spatial distribution of CF and thus affect the 1D1R device performance, which has been discussed in the Figs S3 and S4 of supporting information. After this electroforming process, the pn heterostructure device shows a typical unipolar RS behavior (Fig. 1(b)). The positive set and reset voltages (V_SET_ and V_RESET_) of ~7 V and ~3 V can cause the reversible switching between the high resistive state (HRS) and the low resistive state (LRS). It is noted that the LRS still shows the rectifying behavior for this heterostructure device (Fig. 1(c)). To clarify the rectification characteristics, we study the conductive behaviors of the HRS and LRS. According to the junction emission model, the current density J can be expressed as: 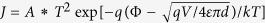, where A*, ε, *Φ*, and d are the Richardson constant, dielectric constant, barrier height and effective switching thickness, respectively^19^,^20^. The relation of V^1/2^ ∝ In(I) indicates that a junction emission mechanism is suitedfor the conductive behaviors of the HRS and LRS (Fig. 1(d)), indicating the existence of the junction. Combining with the above analysis (Figs S1 and S2), the junction emission is attributed to the p^+^-Si/n-ZnO diode. Thus, the p^+^-Si/n-ZnO/Al device works as one diode in series with one unipolar RRAM device, realizing the 1D1R switching. Compared with the traditional 1D1R devices with discrete components (one diode in series with one unipolar RRAM), this simple structure device can effectively simplify the fabrication process and increase the success rate in the integrated crossbar network. Meanwhile, the single vertical structure can also decrease the spatial size, potentially increases the 3D integration density.

**Figure 2:**
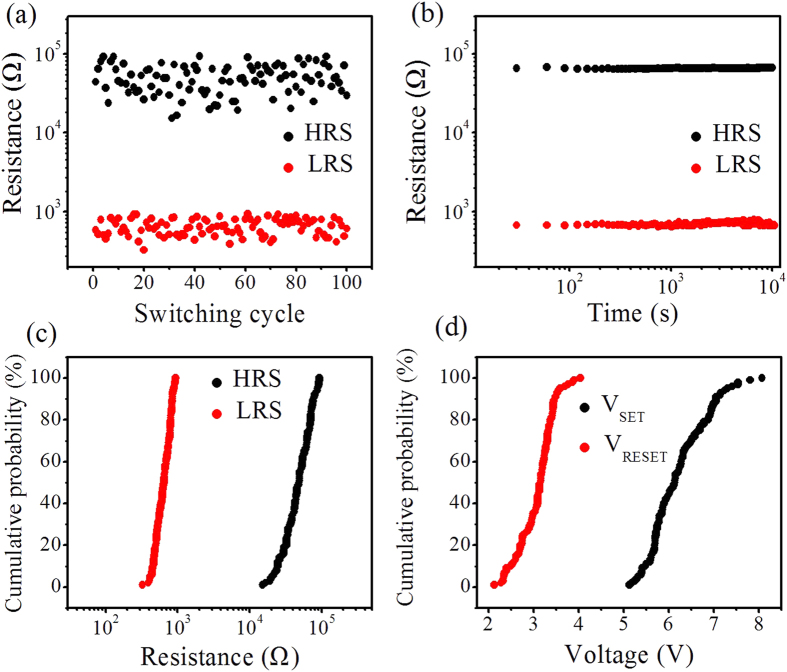
(**a**) The HRS/LRS distribution of 100 repeated RS cycles of the 1D1R device. (**b**) The retention property of the HRS and LRS. (**c**) Cumulative probability plots from (**a**) for the HRS and LRS. (**d**) Cumulative probability for the set and reset voltage. The RS behaviors were tested at room temperature.

The reliability of this 1D1R device is demonstrated in its cycling endurance and retention time tests, as shown in Fig. 2(a) and (b), respectively. The resistance states scatter to a certain extent during the unipolar RS, and the resistance ratios of HRS and LRS approach 100 in 100 continuous sweeping cycles, (see Fig. 2(a)). Meanwhile, both the LRS and HRS can be retained for over 10^4^ s without any degradation. Herein, a 1V bias is used as the readout voltage for the 1D1R device, because its LRS I-V curve indicates that the turn-on voltage of p^+^-Si/n-ZnO diode is less than 1V (Fig. 1(c)). The resistances of HRS and LRS of this 1D1R device are uniform, as shown in Fig. 2(c). A larger “window” of more than 1 V difference between V_SET_ and V_RESET_ ensures error-free RS operation (Fig. 2(d)). In addition, it can be seen from the LRS I-V curve in Fig. 1(c) that the 1D1R device shows a high rectification ratio of >10^3^ at ±1 V, and especially its reverse resistance is about 10^6^ Ω at −1 V, which is one order of magnitude higher than the HRS resistance. The high reverse resistance of p^+^-Si/n-ZnO diode ensures an effective suppression of sneak current even in the worst case, where the selected memory cell remains in the HRS and several neighboring cells all are in the LRS. Therefore, the single-stacked 1D1R device has great potential application in the crossbar network.

**Figure 3:**
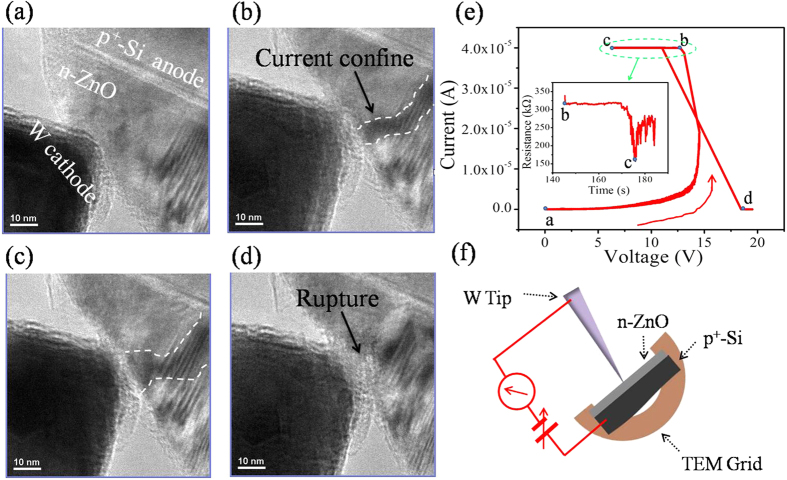
A series of *in-situ* TEM images clipped from the video and the corresponding I-V measurements. (**a**) The initial state. (**b**) The columnar CF have formed when the device switch to the LRS. (**c**) The CF becomes larger in diameter by the applied voltage. (**d**) The CF rupture once the device switch to the HRS. (**e**) Corresponding I-V curve of the RS process. The inset shows the variationas the time when the device reaches the CC. (**f**) The schematic diagram of *in-situ* TEM device.

How to operate the RS in p^+^-Si/n-ZnO/Al heterostructure devic? To understand the mechanism, we focus on the evolution of CF during the RS process and analyze the CF’s components by *in-situ* TEM. Here, the schematic diagram of *in-situ* cross-sectional TEM device was shown in Fig. 3(f). Figure 3 shows the forming/rupture of the CF by *in-situ* observations and the corresponding I-V measurements. The ZnO film shows the initial high resistance value (see Fig. 3(a)) and is electroformed by using a voltage sweep process with a CC. When the voltage is gradually raised to about 13V, it causes a switching from the HRS to LRS. It is known that, in many transition metal oxides, oxygen ion defects and oxygen vacancies are much more mobile than cations under an external electric field^21^. The oxygen ions migration due to the electric field could cause the oxygen-vacancy doping, resulting in the decrease of the resistance^2^,^22^. Thus, the switching behavior could be attributed to the accumulation of oxygen vacancy and formation of the CF in the ZnO film (Fig. 3(b)). As the continuous effect of the electric field, the resistance gradually decreases (the inset of Fig. 3(e)) and the CF become larger in diameter (Fig. 3(c)). We also find that the resistive value fluctuates as the applied voltage, which may be due to the competition of CF’s forming and rupture in the ZnO film^23^,^24^. Then the current suddenly drops as a typical unipolar RS, the sample switches to the HRS (Fig. 3(e)) and the rupture of CF is observed (Fig. 3(d)). The forming and rupture processes of CF were also recorded in Movie S1 of the supporting information. The experiment directly indicated that the RS behavior of p^+^-Si/n-ZnO/Al RRAM is associated with the forming and rupture of the CF due to the migration, accumulation and diffusion of oxygen vacancies.

**Figure 4:**
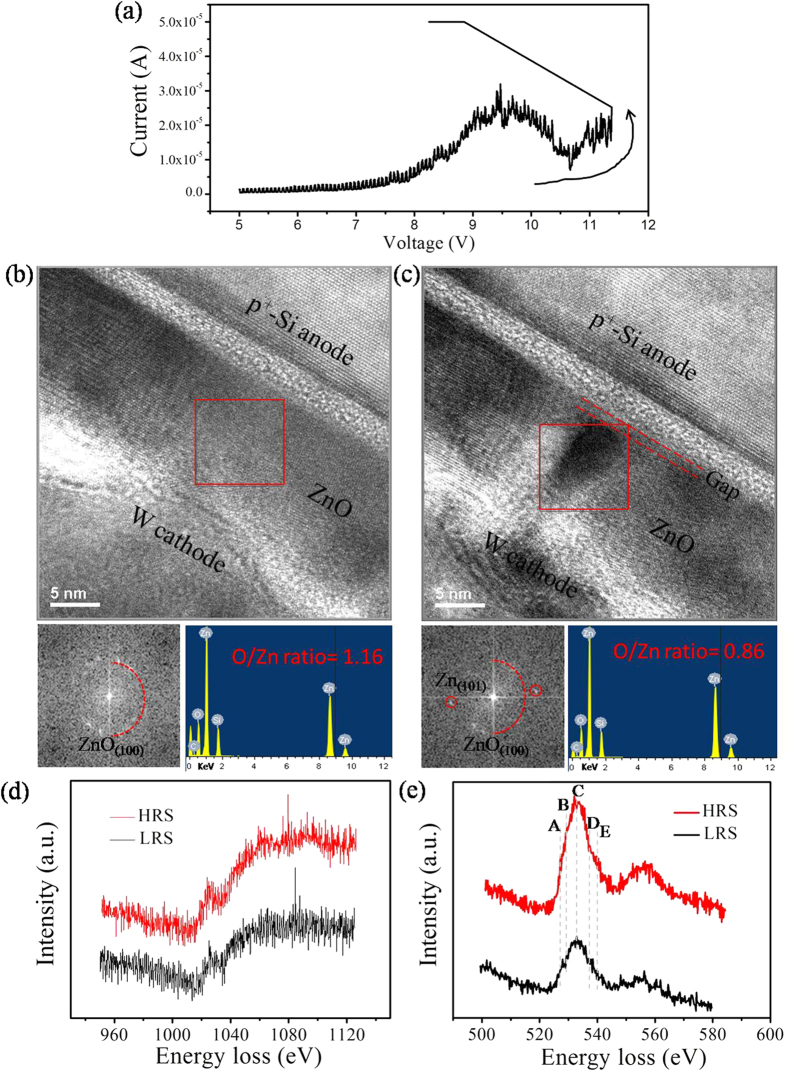
(**a**) The I-V forming curve from the HRS to the LRS in another *in-situ* TEM sample. (**b**) and (**c**) The TEM images corresponding the HRS and the LRS, respectively. Their corresponding FFT pattern and EDS spectra are shown in the lower left and the lower right insets, respectively. (**d**) and (**e**) The EELS spectrum of the Zn L-edge and O K-edge, respectively.

We further analyze the CF’s components of the *in-situ* specimen through the TEM images, EDS and EELS spectrum. The forming of CF was recorded in Movie S2 of the supporting information. The initial TEM image of ZnO film is acquired from another sample (Fig. 4(b)). A voltage (~11.5 V) triggers the abrupt increase of current to the CC, and the resistance switches from the HRS to LRS, as shown in Fig. 4(a). Meanwhile, the CF appears in the LRS of this 1D1R device (Fig. 4(c)). Then, we contrast and analyze the HRS and LRS region (red square marked in Fig. 4(b) and (c), respectively). The Fast Fourier Transform (FFT) results of HRS and LRS are shown in the lower left insets of Fig. 4(b) and (c), respectively. The diffraction rings indicate that the ZnO film has a polycrystalline structure in the HRS. The diffraction spot marked with a red-line circle in the lower left insets of Fig. 4(c) demonstrates that the LRS may exist in a newly generated Zn atom phase, revealing that the CF is converted to ZnO_1−x_. This result indicates that the CF is an oxygen-deficient (oxygen vacancy) states, which has been further proved by measuring the EDS spectra and EELS spectra. The atomic ratio of oxygen and zinc in the HRS is higher than that in the LRS, as shown in the lower right insets of Fig. 4(b) and (c), respectively. In both the HRS region and LRS region, the similar Zn L-edge signals are shown in the Fig. 4(d), matched with the EELS spectra of ZnO^25^,^26^,^27^. But here shows the difference of the OK-edge signal between the CF and ZnO matrix. The intensities of the B and C peaks of CF region are found to decrease as compared to that of ZnO matrix (Fig. 4(e)). The reduction in intensity is addressed to a decrease of the available empty O 2p states, which implies the out-diffusion of oxygen from the CF. Furthermore, the E peak is not visible in the CF region, suggesting the occurrence of structural disorder, which is due to the presence of a large quantity of oxygen vacancies^27^,^28^. Due to the high vacuum in the TEM chamber making the re-oxidation of the oxygen-deficient filament difficult, the reset process under the vacuum often failed^29^. Compared with the *ex-situ* RS test, we found that it is more difficult to realize the reset process in our *in-situ* TEM experiment. Thus, it implies that the oxygen vacancy CF has formed in the ZnO film.

**Figure 5:**
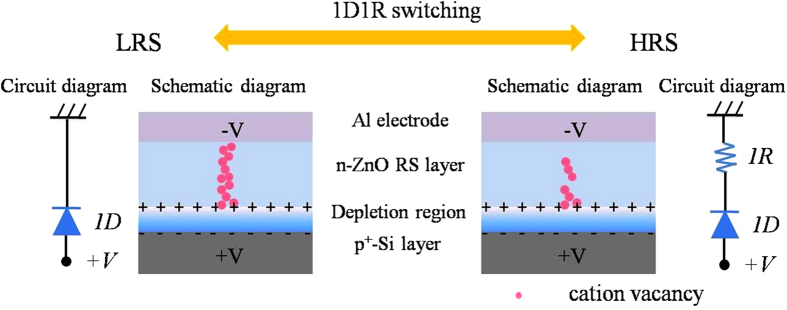
The schematic diagrams illustrating the morphology and distribution of CF for the LRS and HRS of p^+^-Si/n-ZnO/Al 1D1R device, and their equivalent circuit.

These studies indicate that the RS behaviors occur in the ZnO layer for the p^+^-Si/n-ZnO/Al device. Many reports and our previous work declared that it did not appear the diode characteristics in the traditional metal-insulator-metal structure of ZnO-based RRAM devices^30^,^31^. Considering the special structure, one diode should be attributed to the Si/ZnO interface in this 1D1R device. A depletion region will appear at the Si/ZnO interface owing to their different Fermi level decided by the energy band. Furthermore, the depletion region and the band bending are mainly located in the n-ZnO layer since the electron concentration of p^+^-Si film is much higher than the hole concentration of ZnO film. Now, let us discuss the RS process in this p^+^-Si/n-ZnO/Al 1D1R device. When a positive sweep voltage is applied to the device, the electric field will simultaneously affect the ZnO dielectric layer and Si/ZnO junction. Then the resistance of Si/ZnO junction will decrease with increasing the sweep voltage due to the decrease of band bending and depletion region width. The applied external electric field will mainly affect the ZnO dielectric layer. The oxygen ions will migrate due to the electric field, and it leads to the accumulation of the oxygen vacancy and forms the CF in the ZnO film. However, in the depletion region, the built-in electric field, which has an opposite direction with respect to the applied external electric field, will inhibit the migration of oxygen ions. The CF is very difficult to form in ZnO depletion region because of the cancellation between the internal and external electric fields. That is, the CF does not pass through the entire ZnO layer, but is only localized to the outside of depletion region, as shown in the schematic diagram Fig. 5. Here, the *in-situ* TEM images also verify our design. The integrated CF through the Al and p^+^-Si electrode is not formed, but the “gap” at the Si/ZnO interface appears. In other words, the CF is only formed at the outside of depletion region for the LRS, as shown in Fig. 4(c). The microstructure of ZnO depletion layer, as well as the quality of Si/ZnO interface, is not destroyed by the CF formation, enabling a stable and good rectifying function. It has been clearly examined by the *ex-situ* tests in the Fig. S5 of supporting information. The LRS state of the 1D1R device can be regarded as the localized CF in series with the pn junction (Circuit diagram of Fig. 5). When another positive voltage is applied, the localized CF will rupture by the Joule heating. The resistance value of HRS should be the sum of the resistance of ZnO film and Si/ZnO diode. The formation and rupture of the localized CF model has been proposed to explain the 1D1R switching in the p^+^-Si/n-ZnO/Al RRAM device.

## Conclusions

In summary, the 1D1R switching behaviors have been obtained in the p^+^-Si/n-ZnO/Al RRAM device. The RS mechanism can be attributed to the formation and rupture of the CF based on the migration of oxygen ions, the CF is composed of the oxygen vacancy by *in-situ* TEM analysis. The TEM characterization demonstrates the existence of the depletion region during the RS process, revealing one Si/ZnO diode in series with one unipolar ZnO-based RRAM model. Compared with the traditional 1D1R devices with discrete components, this single-stacked structure can effectively simplify the fabrication process and potentially increase the 3D integration. For the next step, the new fabrication technology of Si or some p-type materials with high conductivity is necessary to construct the single-stacked 1D1R device.

## Methods

### Device Fabrication

#### *Ex-situ* device

The substrates for film deposition were heavily doped p-type silicon purchased from MTI Corporation, with A low resistivity of ~0.005 Ω · cm. The ZnO film was grown on p^+^-Si substrates by pulsed laser deposition, and the growth was performed in 20 Pa pure O_2_ atmosphere at 600 °C. Finally, the Al electrode was thermally evaporated on the top to complete the device fabrication.

#### *In-situ* device

The W tips for *in-situ* measurements were made using a homemade electrochemical corrosion cell using KOH solution electrolyte. The tips were typically 10 nm at the sharpest end. All the TEM cross-sectional devices were prepared by conventional mechanical polishing and argon ion milling. Then this sample was loaded into a homemade specimen holder. We estimated the thickness of the interesting areas of the devices to be about 30 nm to 60 nm, using electron energy loss spectroscopy (EELS) analyzed by Digital Micrograph software.

#### Device Test

For all measurements, we define that the positive current flows from p^+^-Si to Al (or W) electrodes.The *ex-situ* measurements were carried out using a semiconductor analyzer Agilent B1500 at ambient environmental conditions. The *in-situ* measurements were conducted in a JEOL 2010F TEM combined with an Agilent B2900 Precision Source/Measure Unit (SMU). An accelerating voltage of 200 kV was used. The TEM holders in our experiments were all homemade and dedicatedly designed for *in-situ* TEM experiments. The W tip was driven by a nanomanipulator as a movable electrode.

## Additional Information

**How to cite this article:** Zhang, L. *et al*. Resistive switching mechanism in the one diode-one resistor memory based on p^+^-Si/n-ZnO heterostructure revealed by *in-situ* TEM. *Sci. Rep.* **7**, 45143; doi: 10.1038/srep45143 (2017).

**Publisher's note:** Springer Nature remains neutral with regard to jurisdictional claims in published maps and institutional affiliations.

## Supplementary Material

Supplementary Movie 1

Supplementary Movie 2

Supplementary Information

## References

[b1] Chung A., Deen J., Lee J. S. & Meyyappan M. Nanoscale memory devices. Nanotechnology 21, 2001–2022 (2010). 10.1088/0957-4484/21/41/412001 20852352

[b2] Strukov D. B., Snider G. S., Stewart D. R. & Williams R. S. The missing memristor found. Nature (London) 453, 80–83 (2008). 18451858 10.1038/nature06932

[b3] Huang P. . A Physics-Based Compact Model ofMetal-Oxide-Based RRAM DC andAC Operations. IEEE Electron Device Lett. 60, 4090–4097 (2013).

[b4] Zhang L. . Oxygen-concentration effect on p-type CuAlO_x_ resistive switching behaviors and the nature of conducting filaments. Appl. Phys. Lett. 104, 3512–3515 (2014).

[b5] Lee M. J. . A fast, high-endurance and scalable non-volatile memory device made from asymmetric Ta_2_O_5−x_/TaO_2−x_ bilayer structures. Nature Mater. 10, 625–630 (2011). 21743450 10.1038/nmat3070

[b6] Jo S. H., Kim K.-H. & Lu W. High-density crossbar arrays based on a Si memristive system. Nano Lett. 9, 870–874 (2009). 19206536 10.1021/nl8037689

[b7] Xia Q. F., Yang J. J., Wu W., Li X. M. & Williams R. S. Self-Aligned Memristor Cross-Point Arrays Fabricated with One Nanoimprint Lithography Step. Nano Lett. 10, 2909–2914 (2010). 20590084 10.1021/nl1017157

[b8] Chang Y. F. . Demonstration of Synaptic Behaviors and Resistive Switching Characterizations by Proton Exchange Reactions in Silicon Oxide. Sci. Rep. 6, 21268–21278 (2016). 26880381 10.1038/srep21268 PMC4754682

[b9] Shin Y. C. . (In,Sn)_2_O_3_/TiO_2_/Pt Schottky-type diode switch for the TiO_2_ resistive switching memory array. Appl. Phys. Lett. 92, 2904–2906 (2008).

[b10] Huang J. J., Kuo C. W., Chang W. C. & Hou T. H. Transition of stable rectification to resistive-switching in Ti/TiO_2_/Ptoxide diode. Appl. Phys. Lett. 96, 2901–2903 (2010).

[b11] Lee M. J. . 2-stack 1D-1R cross-point structure with oxide diodes as switch elements for high density resistance RAM applications. Tech. IEEE Int. Electron Devices Meeting, 771–774 (2007).

[b12] Seo J. W. . A ZnO cross-bar array resistive random access memory stacked with heterostructure diodes for eliminating the sneak current effect. Appl. Phys. Lett. 98, 3505–3507 (2011).

[b13] Zhang F. F. . Rectifying characteristics and implementation of n-Si/HfO_2_ based devices for 1D1R-based cross-bar memory array. Silicon Nanoelectronics Workshop, 1–2 (2012).

[b14] Wei J. K., Xu Z., Wang H., Wang W. L. & Bai X. D. *In-situ* TEM study of the dynamic behavior of the graphene-metal interface evolution under Joule heating. Sci China Tech Sci 59, 1080–1084 (2016).

[b15] Chen J. Y. . Dynamic Evolution of Conducting Nanofilament in Resistive Switching Memories. Nano Lett. 13, 3671–3677 (2013). 23855543 10.1021/nl4015638

[b16] Tian X. Z. . Bipolar Electrochemical Mechanism for Mass Transfer in Nanoionic Resistive Memories. Adv. Mater. 26, 3649–3654 (2014). 24634096 10.1002/adma.201400127

[b17] Park G. S. . *In situ* observation of filamentary conducting channels in an asymmetric Ta_2_O_5−x_/TaO_2−x_ bilayer structure. Nat. Commun. 4, 2382–2390 (2013). 24008898 10.1038/ncomms3382

[b18] Wei J. K., Jiang N., Bai X. D. & Liu J. Y. Strong Coupling between ZnO Excitons and Localized Surface Plasmons of Silver Nanoparticles Studied by STEM-EELS. Nano Lett. 15, 5926–5931 (2015). 26237659 10.1021/acs.nanolett.5b02030

[b19] Zhang L. . Localized resistive switching in a ZnS-Ag/ZnS double-layer memory. J. Phys. D: Appl. Phys. 47, 5101–5106 (2014).

[b20] Zhong L., Jiang L., Huang R. & Groot C. H. Nonpolar resistive switching in Cu/SiC/Au non-volatile resistive memory devices. Appl. Phys. Lett. 104, 3507–3511 (2014).

[b21] Waser R. & Aono M. Nanoionics-based resistive switchingmemories. Nature Mater. 6, 833–840 (2007). 17972938 10.1038/nmat2023

[b22] Yang J. J. . Memristive switching mechanism for metal/oxide/metal nanodevices. Nat. Nanotechnol. 3, 429–433 (2008). 18654568 10.1038/nnano.2008.160

[b23] Chang T. S., Jo H. & Lu W. Short-Term Memory to Long-Term Memory Transition in a Nanoscale Memristor. ACS nano 5, 7669–7676 (2011). 21861506 10.1021/nn202983n

[b24] Zhang L. . Coexistence of bipolar and unipolar resistive switching behaviors in the double-layer Ag/ZnS-Ag/CuAlO_2_/Pt memory device. Appl. Surf. Sci. 360, 338–341 (2016).

[b25] Chang G. S. . Effect of Co and O defects on the magnetism in Co-doped ZnO: Experiment and theory. Phys. Rev. B 75, 5215–5220 (2007).

[b26] Wang R. M., Xing Y. J., Xu J. & Yu D. P. Fabrication and microstructure analysis on zinc oxide nanotubes. New J. Phys. 5, 115.1–115.7 (2003).

[b27] Ding Y. & Wang Z. L. Electron energy-loss spectroscopy study of ZnO nanobelts. J Elect. Micros. 54, 287–291 (2005). 10.1093/jmicro/dfi039 16123068

[b28] Guglieri C. & Chaboy J. O. K-Edge X-ray Absorption Spectroscopy in Al-Doped ZnO Materials: Structural vs Electronic Effects. J. Phys. Chem. C. 118, 25779–25785 (2014).

[b29] Chen J. Y. . Switching Kinetic of VCM-Based Memristor: Evolution and Positioning of Nanofilament. Adv. Mater. 27, 5028–5033 (2015). 26193454 10.1002/adma.201502758

[b30] Chang W. Y. . Unipolar resistive switching characteristics of ZnO thin films for nonvolatile memory applications. Appl. Phys. Lett. 92, 2110–2112 (2008).

[b31] Wang Z. Q., Xu H. Y., Zhang L., Ma J. G. & Liu Y. C. Performance improvement of resistive switching memory achieved by enhancing local-electric-field near electromigrated Ag-nanoclusters. Nanoscale 5, 4490–4994 (2013). 23584667 10.1039/c3nr33692a

