# Supplementary material for: Resistive switching mechanism in the one diode-one resistor memory based on p+-Si/n-ZnO heterostructure revealed by in-situ TEM
Source: Sci Rep. 2017 Mar 21;7:45143. doi: 10.1038/srep45143 (PMC5359668; doi:10.1038/srep45143)
Supplement: Supplementary Information [file srep45143-s3.pdf]

SUPPORTING INFORMATION

Resistive switching mechanism in the one diode-one resistor memory based on p^+-Si/n-ZnO heterostructure revealed by in-situ TEM

Lei Zhang[1], Liang Zhu[1], Xiaomei Li[1], Zhi Xu[1], Wenlong Wang[1], & Xuedong Bai[1, 2, 3]*

[1]*Beijing National Laboratory for Condensed Matter Physics and Institute of Physics, Chinese Academy of Sciences, Beijing 100190, China*

[2]*Collaborative Innovation Center of Quantum Matter, Beijing 100190, China*

[3]*School of Physical Sciences, University of Chinese Academy of Sciences, Beijing 100190, China*

**Corresponding Author, Email: xdbai@iphy.ac.cn*

Captions of Movie S1 and S2

Movie S1 The composition of in-situ TEM images. The forming and rupture of the CF were observed about 6s and 9s under an external electric field (corresponding I-V measurements in Fig. 3(e)), respectively.

Movie S2 The composition of in-situ TEM images. The forming of the CF was observed under an external electric field (corresponding I-V measurements in Fig. 4(a)).

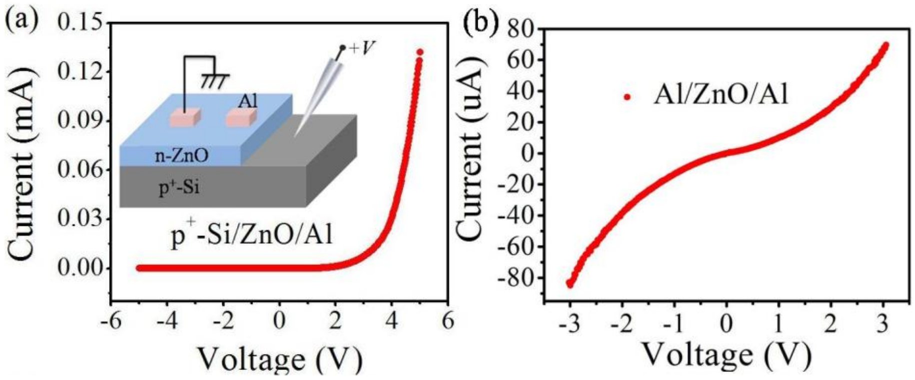

Figure S1 (a) The I-V cures of p$^+$-Si/n-ZnO/Al 1D1R device. Structure diagram of this device is shown in the inset of Fig. S1 (a). (b) The I-V cures of Al/ZnO/Al reference device.

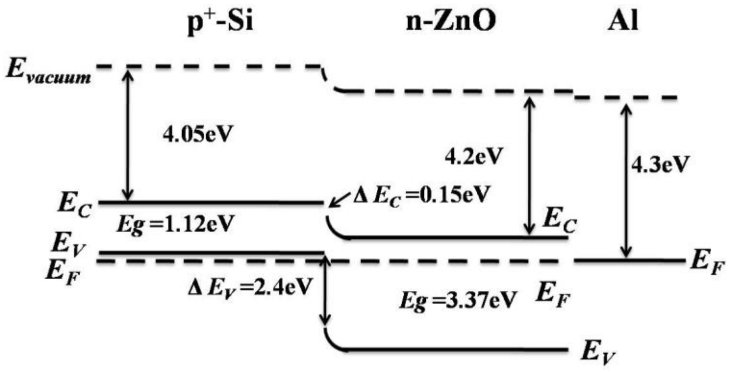

Figure S2 The band diagram of p$^+$-Si/n-ZnO/Al 1D1R device.

In order to justify the existence of a p-n junction, the I-V curves of p$^+$-Si/n-ZnO/Al 1D1R device and Al/ZnO/Al reference device were measured in Fig. S1 (a) and (b), respectively. The p$^+$-Si/n-ZnO/Al device shows the rectification characteristic. The Al/ZnO/Al reference device exhibits the symmetric I-V curve and smaller junction resistance compared with the p$^+$-Si/n-ZnO/Al device, which indicates that the junction is mainly source from the p$^+$-Si/n-ZnO diode. The energy band of p$^+$-Si/n-ZnO/Al device further illuminates that the rectification characteristic is source from the p$^+$-Si/n-ZnO diode (see Fig. S2).

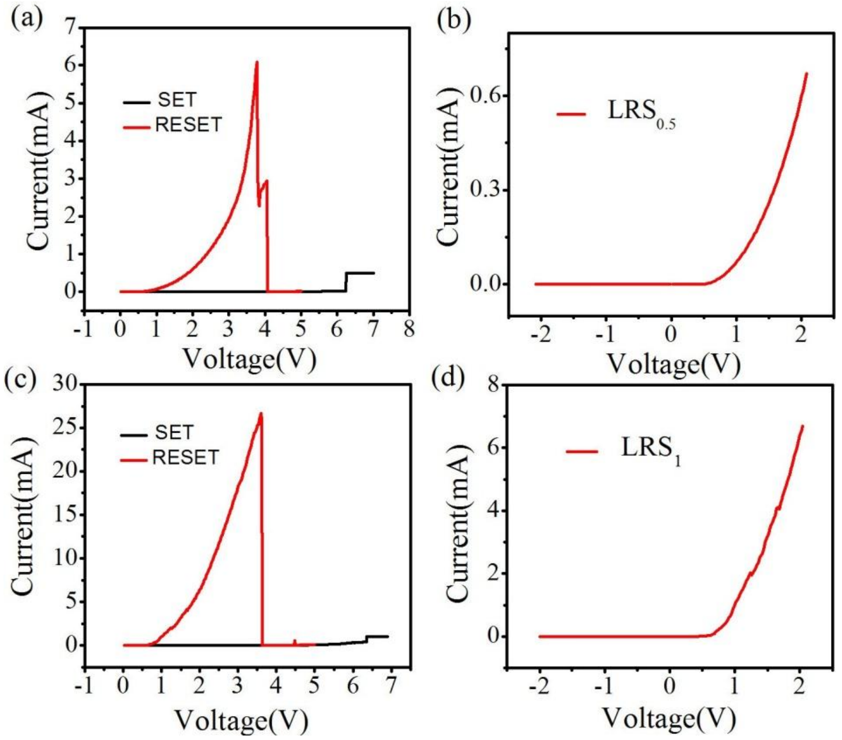

Figure S3 (a) and (c) The RS curves with 0.5 and 1 mA CCs, respectively. (b) and (d) The I-V curve of their $LRS_{0.5}$ and LRS_1, respectively.

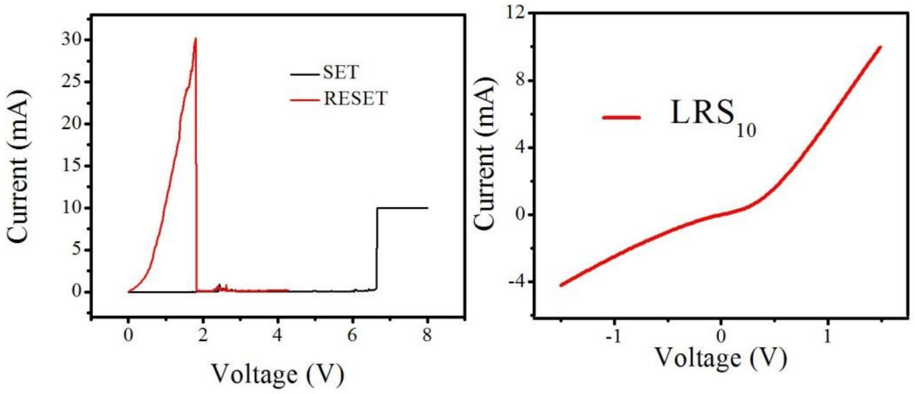

Figure S4 The RS curves with 10 mA CCs, and the I-V curve of the LRS_{10}.

Here, we have also tested the resistive switching (RS) behaviors with different current compliance (CC) (see Fig. S3 and S4). The LRS with 0.5 and 1mA CC ($LRS_{0.5}$ and LRS_1) show the nonsymmetric rectifying characteristics, which indicates that the pn junction still exists. It indicates that the p^+-Si/n-ZnO/Al device can realize the 1D1R switching with low CC. Once increasing the CC to 10mA, the diode characteristics of the LRS (LRS_{10}) will be degraded, and it seems to form the

integrated conducting filament (CF) in the ZnO film, as shown in Fig. S4. The high CC is expected to destroy the pn junction. The LRS resistance gradually increases with the decrease of the CC. It can be seen that the level of CC may change the spatial distribution of CF and thus affect the 1D1R device performance. Therefore, the proper CC is very important to enhance the endurance and reliability of our single-stacked 1D1R memory.

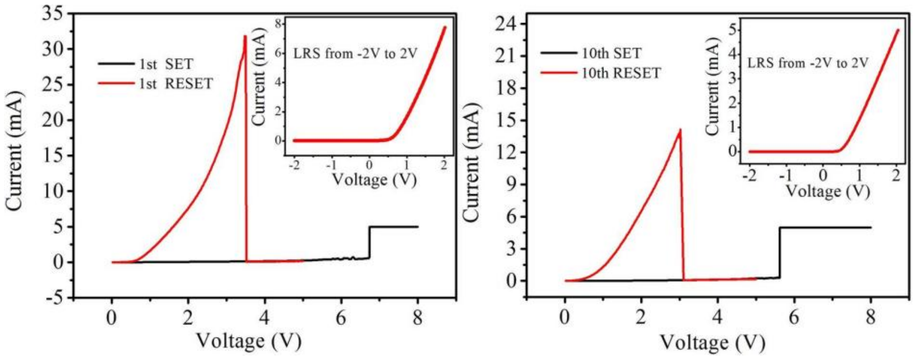

Figure S5 The I-V curves of the 1st and 10th RS cycle. Their insets show the rectifying I-V curves for the LRS.

The continuous ex-situ RS cycle can be obtained in the p^+-Si/n-ZnO/Al 1D1R device. Here, the 1st and 10th RS behaviors were shown in Fig. S5(a) and (b), respectively. We can see that the LRS shows the asymmetric rectifying I-V characteristics during the continuous switching cycle. It indirectly indicates that the junction still exists at the p^+-Si/n-ZnO interface during the RS.
